# Supplementary material for: The Physiology of Postharvest Tea (Camellia sinensis) Leaves, According to Metabolic Phenotypes and Gene Expression Analysis
Source: Molecules. 2022 Mar 5;27(5):1708. doi: 10.3390/molecules27051708 (PMC8911848; doi:10.3390/molecules27051708)
Supplement: Supplementary file 1 [file molecules-27-01708-s001.zip › molecules-1607095-supplementary.pdf]

Table S1. PCR primers used for gene expression analysis.

Enzyme name	Primer	Primer sequence (5'-3')
Farnesene synthase	qCsFAS-F	GAGATAGCCAGGAAGAAG
	qCsFAS-R	TGGTAAATAAAATGAGCC
Limonene synthase	qCsLMS-F	GTTGACAGATGGGATGTG
	qCsLMS-R	CAATGTTGGCTTGTACCC
Germacrene D synthase	qCsGDS-F	GGTACCGGAGGTCACTCG
	qCsGDS-R	TGTCATCATGGTGATCAT
Mevalonate-5-pyrophosphate decarboxylase	qCsMVD-F	GGAATCGCTCAGAAGAAT
	qCsMVD-R	TTAGGAGGGAAGTGGAAA
4-Hydroxy-3-methylbutenlyl diphosphate reductase	qCsHDR-F	GGGGTTTGATCCAGACAG
	qCsHDR-R	TGAAGTGCTCGTTGAGGT
Terpene synthase 78	qCsTPS78-F	AGGCATTGATTACCACTTCCGGGAG
	qCsTPS78-R	GCACATTGTAGCCTTGTTGCCTCAA
Terpene synthase 77	qCsTPS77-F	GCAAGAAGGGTACAATGTGCCCGCG
	qCsTPS77-R	CTGCGGCTTGATCAAGAATGTCTTC
Tea caffeine synthase1	qCsTCS1-F	TTACTTTTCTGACGAGGCA
	qCsTCS1-R	TGCGTTAAGAGCTTGAAGG
Tea caffeine synthase2	qCsTCS2-F	CCATTGCAGTTGGCACCA
	qCsTCS2-R	ACAACCCAAGTCCACTGC
Alanine decarboxylase	qCsAlaDC-F	GATTGGTTTGCCCGTCTATG
	qCsAlaDC-R	ATTATGGCTGGTTTGTCCTT
Theanine synthetase	qCsTSI-F	GTTGATGTTTCTGGGCAGCA
	qCsTSI-R	CTCACCCACACCAGTCAGAT
Glutamine synthetase 2	qCsGS2-F	CAGATTTTGGCTCCTTCC
	qCsGS2-R	GTGCTGTTTTCGGACTGT
Flavonoid 3′,5′-hydroxylase	qCsF3'5'H-F	AAAGTAATTGGAAGAAACCGCC
	qCsF3'5'H-R	TAGGAATACTGAGGGGAACTGA
Flavonoid 3-hydroxylase	qCsF3H-F	GACCTACTTCTCATACCCGATC
	qCsF3H-R	AGTCCATCAATTTCTCGCTGTA
Leucoanthocyanidin reductase	qCsLAR-F	GTGTTGGAATCTGTGTCCGCAG
	qCsLAR-R	CTCCATGAATGCTTGATCCTTG
Anthocyanidin reductase	qCsANR-F	AACCAGCAATTCAAGGAGTAGT
	qCsANR-R	TCCCATTGAGCTTATTGATCGA
Serine carboxypeptidase-like acyltransferases 7	qCsSCPL1A7-F2	CGCGCCAATATACTATATGTAGAT
	qCsSCPL1A7-R2	ATAAGAATCACCACCAATGTATAG
Actin	qCsActin-F	GCCATATTTGATTGGAATGG
	qCsActin-R	GGTGCCACAACCTTGATCTT

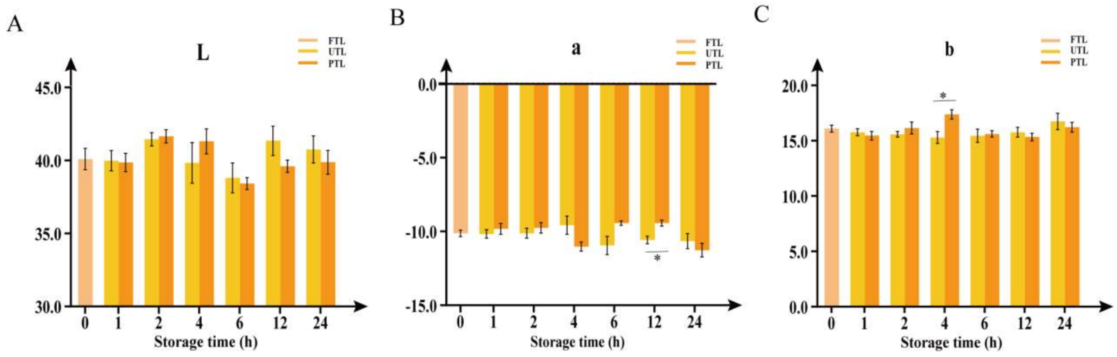

Figure S1. Changes in the values of L* (A), a* (B), and b* (C) in tea leaves in UTL and PTLat different time points and FTL at 0 h. Dunnett's multiple comparisons and t test was used to identify significant differences (∗, $P < 0.05$). Values are the mean±SEM of all replicates.

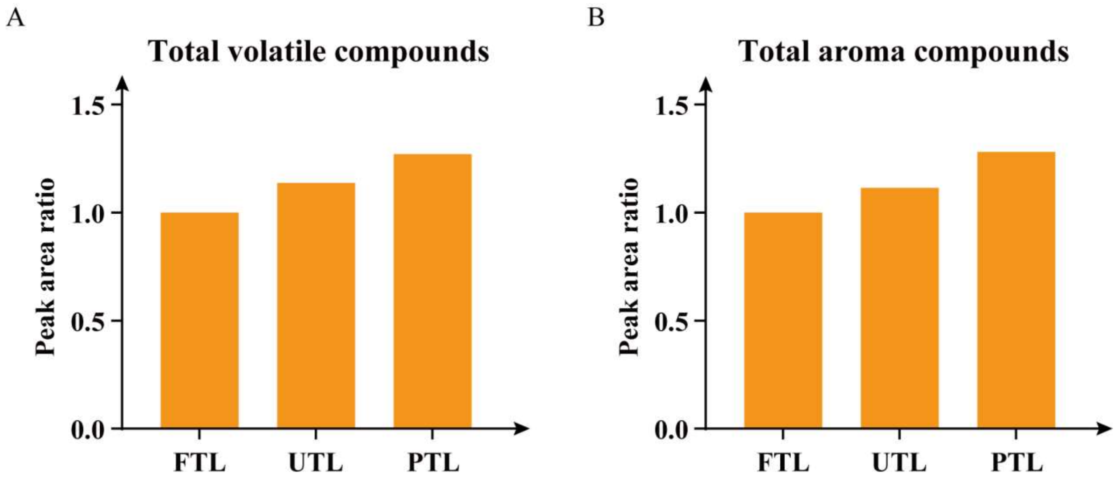

Figure S2. Total volatile compounds (A) and aroma compounds (B) contents in FTL, UTL, and PTL at 24 h postharvest.
